# P-1106. Sulopenem is Efficacious in a Rabbit Model of Inhalational Anthrax

**DOI:** 10.1093/ofid/ofae631.1294

**Published:** 2025-01-29

**Authors:** Sailaja Puttagunta, Steven I Aronin, Stephanie Halasohoris, Ashley Babyak, Jade Spencer, Annette Gray, Marjorie Torres, Christopher Klimko, Christopher Cote, Mary Hourihan, James Meinig

**Affiliations:** Iterum Therapeutics, Old Saybrook, Connecticut; Iterum Therapeutics, Old Saybrook, Connecticut; U.S. Army Medical Research Institute of Infectious Diseases, Fort Detrick, Maryland; U.S. Army Medical Research Institute of Infectious Diseases, Fort Detrick, Maryland; U.S. Army Medical Research Institute of Infectious Diseases, Fort Detrick, Maryland; U.S. Army Medical Research Institute of Infectious Diseases, Fort Detrick, Maryland; U.S. Army Medical Research Institute of Infectious Diseases, Fort Detrick, Maryland; U.S. Army Medical Research Institute of Infectious Diseases, Fort Detrick, Maryland; U.S. Army Medical Research Institute of Infectious Diseases, Fort Detrick, Maryland; U.S. Army Medical Research Institute of Infectious Diseases, Fort Detrick, Maryland; U.S. Army Medical Research Institute of Infectious Diseases, Fort Detrick, Maryland

## Abstract

**Background:**

Sulopenem (SUL) is a penem β-lactam antibiotic in development for the treatment of resistant bacterial infections. It is available as intravenous and oral prodrug formulations, and its activity aligns with the most urgent drug-resistant antimicrobial threats defined by the CDC. SUL possesses potent activity against Enterobacterales species that encode ESBLs or AmpC-type β-lactamases that confer resistance to 3rd generation cephalosporins. SUL has also demonstrated potent *in vitro* activity against numerous biothreat pathogens, including *Bacillus anthracis* at concentrations likely to be achieved after oral dosing in humans. *B. anthracis* is a CDC Tier 1 Select Agent, and remains of considerable concern for biodefense. The potential for mass casualties resulting from a biothreat incident combined with the risk of antibiotic resistance necessitates development of novel medical countermeasures (MCM) for *B. anthracis*. Orally administered SUL was previously shown to be protective in the murine model of inhalational anthrax and provided proof-of-concept to advance the drug into the well-established rabbit model.

**Figure:**
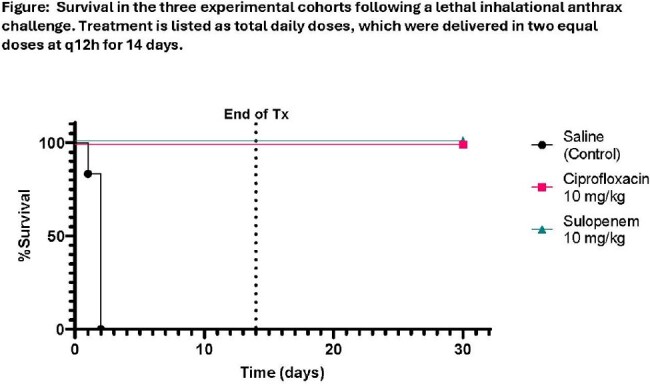

**Methods:**

Female New Zealand White rabbits were challenged with a lethal dose of *B. anthracis* Ames spores via the inhalational route. Post-exposure prophylaxis (PEP) was initiated at 24 ±1 h postchallenge with cohorts (N=6) of animals receiving vehicle (saline, SC), ciprofloxacin (CIP) 10 mg/kg, SC or SUL 10 mg/kg, SC. This dosing regimen was informed by pharmacokinetic models to establish a human-equivalent dose. Treatment was continued BID for 14 days. Clinical progression and survival were assessed up to 30 days postchallenge.

**Results:**

The untreated saline control group demonstrated 100% lethality with a median time to death of 48 h. Both the SUL and CIP groups demonstrated 100% survival through study termination on day 30.

**Conclusion:**

As a MCM for inhalational anthrax, SUL would provide considerable advantages as an addition to the slate of current standards of care. Combined with positive efficacy results in two preclinical models of inhalational anthrax and advantages offered by an orally available penem antibiotic, SUL remains a promising candidate for advanced development for treating *B. anthracis*.

**Disclosures:**

**Sailaja Puttagunta, MD**, Iterum Therapeutics: Employee|Iterum Therapeutics: Employee|Iterum Therapeutics: Stocks/Bonds (Public Company)|Iterum Therapeutics: Stocks/Bonds (Public Company) **Steven I. Aronin, MD**, Iterum Therapeutics: Employee|Iterum Therapeutics: Employee|Iterum Therapeutics: Stocks/Bonds (Public Company)|Iterum Therapeutics: Stocks/Bonds (Public Company)

